# Gas Atomization of Duplex Stainless Steel Powder for Laser Powder Bed Fusion

**DOI:** 10.3390/ma16010435

**Published:** 2023-01-03

**Authors:** Chengsong Cui, Felix Stern, Nils Ellendt, Volker Uhlenwinkel, Matthias Steinbacher, Jochen Tenkamp, Frank Walther, Rainer Fechte-Heinen

**Affiliations:** 1 Leibniz-Institute for Materials Engineering—IWT, Badgasteiner Straße 3, 28359 Bremen, Germany; 2 Chair of Materials Test Engineering (WPT), TU Dortmund University, Baroper Straße 303, 44227 Dortmund, Germany; 3 Faculty of Production Engineering, University of Bremen, Badgasteiner Straße 1, 28359 Bremen, Germany; 4 MAPEX Center for Materials and Processes, University of Bremen, 28359 Bremen, Germany

**Keywords:** laser powder bed fusion, duplex stainless steel, gas atomization, powder property

## Abstract

Duplex stainless steel powders for laser additive manufacturing have not been developed extensively. In this study, the melts of a super duplex stainless steel X2CrNiMoCuWN25-7-4 (AISI F55, 1.4501) were atomized with different process gases (Ar or N_2_) at different atomization gas temperatures. The process gas N_2_ in the melting chamber leads to a higher nitrogen dissolution in the steel and a higher nitrogen content of the atomized powders. The argon-atomized powders have more gas porosity inside the particles than the nitrogen-atomized powders. In addition, the higher the atomization gas temperature, the finer the powder particles. The duplex stainless steel powders showed good processability during PBF-LB/M (Laser powder bed fusion). The gas entrapment in the powder particles, regardless of the gas chemistry and the gas content, appears to have a negligible effect on the porosity of the as-built parts.

## 1. Introduction

Additive manufacturing (AM) is a technology for the manufacturing of complex components layer by layer via a 3D geometric model embedded in computer-aided design (CAD) software [1,2,3,4]. Laser powder bed fusion (PBF-LB/M), also known as selective laser melting (SLM), is one of the additive manufacturing processes where a high-power laser beam is used to selectively melt and fuse metallic particles together once a thin layer of powder has been evenly distributed onto a substrate plate or on the previous layers. This is a very attractive process that is being implemented in both research and industry because it can not only create custom properties but also reduce material usage and give more degrees of freedom with designs, as compared to conventional manufacturing processes.

Duplex stainless steels, with a combination of high mechanical properties and excellent corrosion resistance, are widely used in the oil and gas industry as well as in the chemical industry [5,6]. These steels have ferrite stabilizers (e.g., chromium, silicon, molybdenum) as well as austenite stabilizers (e.g., carbon, nickel, nitrogen). This leads to a duplex microstructure (nearly equal amounts of ferrite and austenite), which provides better corrosion resistance, particularly chloride stress corrosion and chloride pitting corrosion, and higher strength than the common austenitic stainless steels such as AISI 304 or AISI 316. 

Laser additive manufacturing of duplex stainless steels has also been of interest in recent years. Two duplex stainless steels (UNS S32750 and UNS S31803) have been studied using the PBF-LB/M process [7,8,9,10]. In contrast to conventionally produced duplex stainless steels, PBF-LB/M-produced samples are nearly fully ferritic. This is due to the extremely high cooling rate (~10^6^ K/s) during PBF-LB/M, which suppresses the transformation from δ-ferrite to austenite. Therefore, post-heat treatment (solution annealing) is needed to restore the duplex microstructure in the parts. Superior mechanical properties such as tensile strength, yield strength and microhardness were achieved, which are much higher than those of other stainless steel grades (austenitic, ferritic or duplex) [11]. The high dislocation density is responsible for the comparably high ultimate strength and low elongation at fracture in the as-built condition. The mechanical properties of the heat-treated samples showed a high ductility due to the recrystallized austenitic-ferritic microstructure [8].

It is known that powder properties play an important role in PBF-LB/M processability. The characteristics of powder, including particle size distribution, particle shape, powder flowability and apparent density, should be considered and optimized to create good quality powder layers during powder spreading. In general, spherical powders have good flowability and spreadability, resulting in powder layers with high density and as-built parts with low porosity [12,13]. Particles in irregular shape show poor flowability due to interlocking mechanisms [14,15]. On the other hand, very fine particles also lead to particle agglomeration and lack of flowability since the interparticle forces, such as the van der Waals forces between the particles, can exceed the gravitational forces [16,17,18]. 

The metallic powders for laser additive manufacturing are commonly produced by gas atomization. The powder properties are considerably influenced by the used gas atomization process and its parameters, such as atomization gas pressure, melt delivery tube diameter and melting temperature [19]. In addition, the atomization gas temperature also affects the particle size and shape [20,21,22]. Using hot gas for atomization increases the sound velocity of the atomization gas and hence the relative velocity between droplets and gas during breakup. As a result, smaller droplets are achieved, which maintain a spherical shape under the action of surface tension. At the same time, the atomization gas flow rate decreases due to the effect of gas compressibility [20]. Meanwhile, the process gas type also has an influence on the atomization process and results in powders with different chemistry and quality. If steel is melted under a nitrogen atmosphere, nitrogen pickup in the steel melt may take place [23]. If the steel melt is atomized with argon, the argon can be encapsulated in the droplets during droplet deformation and breakup and lead to hollow particles. The aforementioned process parameters and process conditions may have an important impact on the powder properties and thereafter, on the PBF-LB/M part quality.

Duplex stainless steel powders for laser additive manufacturing have not been developed extensively. The powder properties and their processability in PBF-LB/M have not been investigated systematically. In this study, a super duplex stainless steel X2CrNiMoCuWN25-7-4 (AISI F55, 1.4501) was selected as an exemplary alloy. The molten metal was atomized with different process gases (Ar or N_2_) at different atomization gas temperatures. The influence of the process gas on the powder properties, as well as on the as-built part quality, was addressed. Particular attention was given to the porosity inside the particles and the as-built parts. They were examined by means of light optical microscopy, XRM (X-ray Micro-tomography) or X-ray microfocus computed tomography (µCT). 

## 2. Materials and Methods

### 2.1. Raw Material

A super duplex stainless steel X2CrNiMoCuWN25-7-4 (AISI F55, 1.4501, UNS S32760) was selected for the gas atomization experiments. The feedstock for gas atomization was delivered from BGH Edelstahl Freital GmbH, Freital, Germany. The chemical composition of the material, determined by optical emission spectrometry (S-OES, ARL 3460), is given in Table 1. This steel has a high content of chromium and molybdenum with addition of copper and tungsten, which gives the steel a high resistance to surface, pitting and crevice corrosion in chloride-containing media. The nitrogen content in the steel is 0.29 mass %, which promotes the solid solution strengthening of austenite and delays the precipitation of brittle intermetallic phases [24].

### 2.2. Gas Atomization

The gas atomization of the duplex stainless steel was carried out on a self-constructed powder plant with a melting capacity of 0.7 L [15,20,21]. The main process parameters are given in Table 2. The feedstock was inductively melted in an enclosed vessel under a nitrogen atmosphere or under an argon atmosphere at an overpressure of 3 kPa. The process gas was supplied from a liquid gas tank with a purity of 99.99%. The steel melt flew through a delivery tube (3 mm diameter) at the bottom of an Al_2_O_3_ crucible and was atomized by means of a convergent-divergent annular close-coupled atomizer (GD26) [21]. The molten metal stream was disintegrated into small metal droplets by a high-velocity gas flow, forming metal particles after rapid solidification. The atomization gas pressure was 1.6 MPa, corresponding to a gas mass flow rate of 590 kg/h for nitrogen and 780 kg/h for argon at room temperature. When the atomization gas was preheated to approximately 330 °C, the gas mass flow rate was reduced to 393 kg/h for nitrogen and 513 kg/h for argon, respectively. The gas consumption is significantly reduced due to expansion of the gas at high temperature, which is a positive effect of using hot gas atomization. To reduce the formation of satellite particles, an additional gas jet system was applied to inhibit the circulation of the solidified fine particles close to the atomization zone [15]. The anti-satellite gas jet pressure was 21 kPa. 

### 2.3. Powder Classification

The gas-atomized powders were first sieved at 200 µm to remove splats and flakes. Subsequently, the fine particles (approximately < 20 µm) were separated by using an air classifier (Multiprocess system 50 ATP, Hosokawa Alpine). Finally, the powders were mechanically sieved at 63 µm (Ultrasonic sieving machine MS400, Russell Finex). 

### 2.4. Powder Analysis

The gas-atomized powders were analyzed in terms of particle size, shape, flow properties, powder density and porosity in particles. The powder fractions of <200 µm (pre-sieved) and 20–63 µm (PBF-LB/M fraction) were used for particle size measurement. For the other powder analyses, only the powder fraction 20–63 µm was used. The powders were dried in an air-circulating furnace at 200 °C for 2 h.

The particle size distribution of the powders was determined with a laser diffraction instrument (Malvern Mastersizer 2000, Malvern, UK). The particle shape of the powders was analyzed using a Morphologi G3 system (Malvern, UK) based on static image. Qualitative particle morphology of the powders was investigated by means of scanning electron microscopy (CAMSCAN CS44, Cambridge, UK). The polished sections of the powder particles were examined using optical microscopy (Axiophot, Zeiss, Jena, Germany). 

To investigate the dynamic flowability of the powders, a measurement of the avalanche angle was conducted with a self-designed device for the powders. This device consists of a rotating powder drum, a light source and a CCD camera (26 frames per second), which were installed on an optical bench [17]. The powder drum, with an inner diameter of 31 mm and a length of 25 mm, covered on both sides with transparent glass, was filled with 25 g of powder. It rotated at a speed of 5 rpm. The camera records pictures of the powder-free surface and the cross-sectional area of powder inside the drum. The avalanche angle, also known as the dynamic angle of repose, is the angle of a linear regression of the free powder surface just before an avalanche starts, measured to a horizontal line. Manual measurements of at least 10 avalanches were performed for all the powder samples. An Fe-based powder with an avalanche angle > 50° is considered to have limited/poor flowability [12]. 

The apparent density of the powders was measured in accordance with ISO 697:1981. Additionally, the tap density of the powder was measured using a JEL STAV II device (J. Engelsmann AG, Ludwigshafen, Germany) after 1250 tap cycles. Hausner ratio *H* was calculated according to

(1) $$H=\frac{\rho_{T}}{\rho_{B}}$$

where $\rho_{T}$ is the tap density of the powder and $\rho_{B}$ is the apparent density of the powder. A Hausner ratio > 1.25 is considered to be an indication of poor flowability [25].

The porosity inside the powder particles was examined using X-ray micro-tomography (Xradia Versa 520, Zeiss, Germany) of the MAPEX Center for Materials and Processes, University of Bremen, Germany. The powder samples were filled in a plastic tube and scanned with 1.56 µm per voxel in 360° rotation scans, using a beam energy of 140 kV, an energy flux of 72 µA, and a Zeiss filter HE2 (Table 3). For each sample, an image volume of approximately 1000 × 1000 × 1000 voxels was available. Reconstruction of the spatial information on the linear attenuation coefficient in the samples was performed using the Zeiss reconstruction software. Thus, a stack of images containing volumetric information on each powder sample was obtained. The information about the varied X-ray absorption due to various material densities is encoded as grey values in the black-and-white images.

In addition, the content of nitrogen of the gas-atomized powders (fraction 20–63 µm) was measured using carrier hot gas extraction (ONH-2000 analyzer from ELTRA GmbH, Haan, Germany).

### 2.5. PBF-LB/M Experiment

To investigate the processability of the powders and the influence of the powder properties on the quality of as-built parts, the four powders (fraction 20–63 µm) were processed by PBF-LB/M (AconityMINI, Aconity GmbH, Herzogenrath, Germany). Cuboid samples with a dimension of 4 × 4 × 5 mm^3^ and cylindrical samples with a diameter of 4 mm and a height of 6 mm were built on a substrate plate (316L stainless steel) with a diameter of 55 mm under nitrogen atmosphere. The cuboid samples were used for optical microscopy, and the cylindrical samples were used for µCT investigation. A simple laser scan strategy with a rotation angle of 67° between successive layers was used for the building jobs. The main process parameters are given in Table 4, which were supposed to be optimal parameters according to the previous parameter study on austenitic stainless steels and duplex stainless steels [26]. The laser scan speed was varied from 600 mm/s to 1000 mm/s to find the influence of laser energy input on the as-built parts.

### 2.6. Characterization of As-Built Parts

The chemical composition of the as-built parts was determined using optical emission spectrometry (OES; model QSG750, OBLF GmbH, Witten, Germany). The macro sections of the cuboids were examined with a Leica MZ16 Stereomicroscope. Moreover, the cylindrical samples were investigated nondestructively by X-ray microfocus computed tomography (µCT) to characterize the influence of powder properties on the resulting density and defect distribution. The samples were scanned on a Nikon XT H 160 CT system with a microfocus of 3 µm, and a maximum acceleration voltage of 160 kV was used. Scanning parameters were chosen as listed in Table 3.

### 2.7. Image Analysis of Porosity

The porosity in the powder particles as well as in the PBF-LB/M samples was quantitatively investigated by reconstructing the 3D volume and afterward using the analysis software VGStudioMax 3.5. Before defect detection, the surface of the investigated material was automatically determined. Due to the high-quality scans of the powder particles from the XRM, the “Only Threshold” algorithm was applied for defect detection, while for the analysis of the PBF-LB/M cylinders, the “VGEasyPore” algorithm led to satisfactory results. 

Further analysis of the XRM and µCT results is possible as information on the volume and surface of every individual pore can be obtained by the software. The size and shape of the pores are described in terms of equivalent pore diameter *d*_eq_ and sphericity *S* by Equations (2) and (3), respectively, with *V* as defect volume and *A* as its corresponding defect surface.

(2) $$d_{\mathrm{eq}}=\sqrt[{3}]{\frac{6V}{\pi }}$$

(3) $$S=\frac{\pi^{1/3}{\left(6V\right)}^{2/3}}{A}$$

## 3. Results and Discussion

### 3.1. Nitrogen and Oxygen in Gas-Atomized Powder

It is known that steel molten under a nitrogen atmosphere has nitrogen pick-up, depending on the partial pressure of nitrogen, the melt temperature and their chemical composition. An empirical formula used for the calculation of nitrogen solubility [*N*] in mass % in steel melts is given by [27]:

(4) $$\left[N\right]=\sqrt{p}\times 10^{k_{s}}$$

(5) $$\begin{array}{l} k_{s}=\frac{-293}{T}-1.16-(\left(\frac{3757}{T}-0.81\right)(0.072\left[C\right]+0.051\left[Si\right]-0.015\left[Mn\right]-0.039\left[Cr\right]-0.0103\left[Mo\right]\;+\\ 0.0093\left[Ni\right]-0.095\left[V\right]-0.0056\left[W\right]-0.059\left[Nb\right]-0.031\left[Ta\right]-0.35\left[O\right]+0.044\left[N\right])+0.5(\frac{5132}{T}-\;\\ 1.48)(0.0215{\left[C\right]}^{2}+0.000005{\left[Mn\right]}^{2}+0.00058{\left[Cr\right]}^{2}+0.00249{\left[V\right]}^{2}+0.00068{\left[Nb\right]}^{2})+\;\\ 0.167\left(\frac{8124}{T}-3.06\right)(-0.0000068{\left[Cr\right]}^{3}-0.00000401{\left[V\right]}^{3})) \end{array}$$

where *T* is the temperature in K and *p* is the partial pressure of nitrogen in bar (1 bar = 100 kPa).

**Figure 1:**
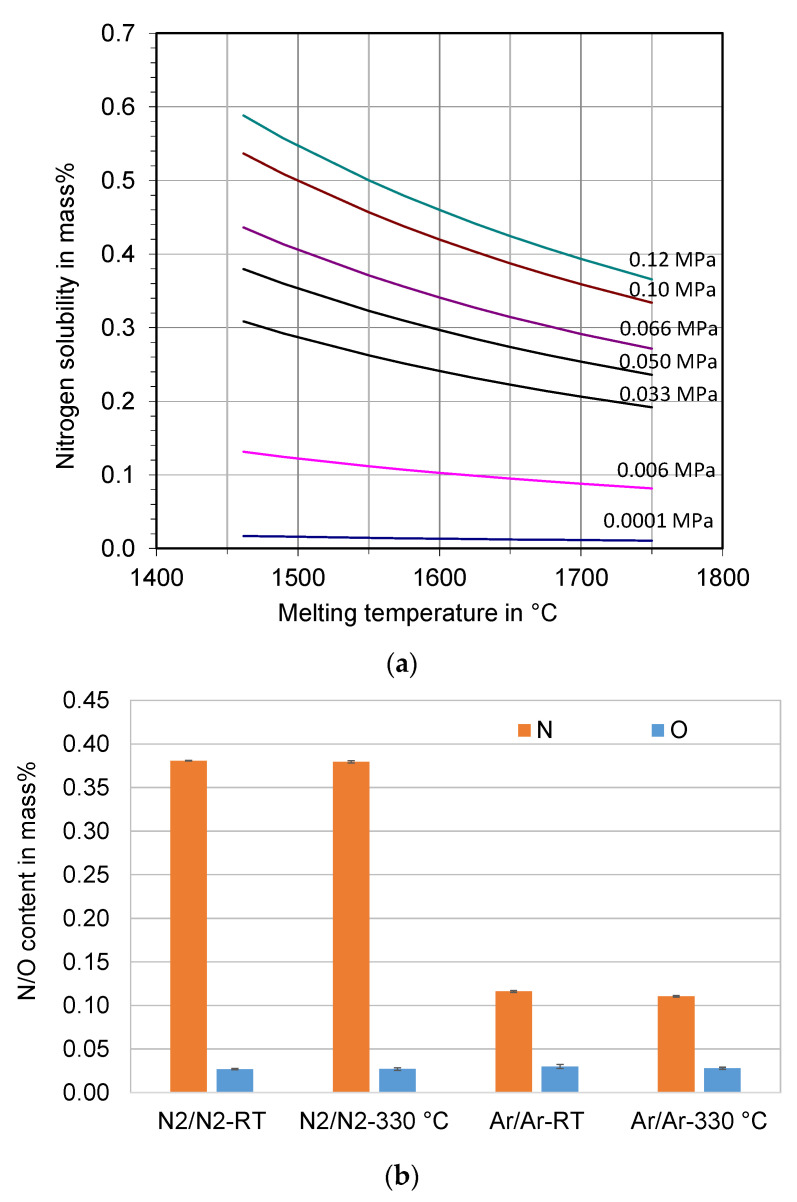
(**a**) Calculated nitrogen solubility in the melt of the super duplex stainless steel X2CrNiMoCuWN25-7-4 at different melt temperatures and nitrogen partial pressures, and (**b**) measured nitrogen and oxygen content of the atomized powders.

The calculated nitrogen solubility in the duplex stainless steel X2CrNiMoCuWN25-7-4 melt is presented in Figure 1a. The nitrogen solubility decreases with increasing melt temperature and decreasing nitrogen partial pressure. For example, it is approximately 3600 ppm (0.36 mass %) at 0.1 MPa N_2_ (ambient pressure) at the melt temperature of 1700 °C, while it is only 110 ppm (0.011 mass %) at 0.0001 MPa N_2_. 

The nitrogen content in the feedstock is 0.29 mass %. It is increased to 0.38 mass % in the atomized powders processed under nitrogen atmosphere, see Figure 1b. The measured nitrogen content of the powders is very close to the calculated value. It indicates that almost all of the dissolved nitrogen in the steel melt remains in the atomized powders. The solidification of the steel melt with primary δ-ferrite, which has a very low solubility for nitrogen [28], did not lead to significant nitrogen outgassing due to the rapid solidification of the atomized droplets. Nitrogen gas entrapment and dissolution in the droplets during atomization may also contribute to a slight increase in the nitrogen content of the powders. The nitrogen content of the powders was 0.11–0.12 mass % when the melting was carried out under an argon atmosphere. There is a significant degassing of nitrogen due to the argon atmosphere.

The oxygen content of all the powders was similar (270–300 ppm). It is in the normal range of gas-atomized steel powders. The atomization gas temperature has no influence on the nitrogen and oxygen content of the powders.

### 3.2. Particle Size and Shape

**Figure 2:**
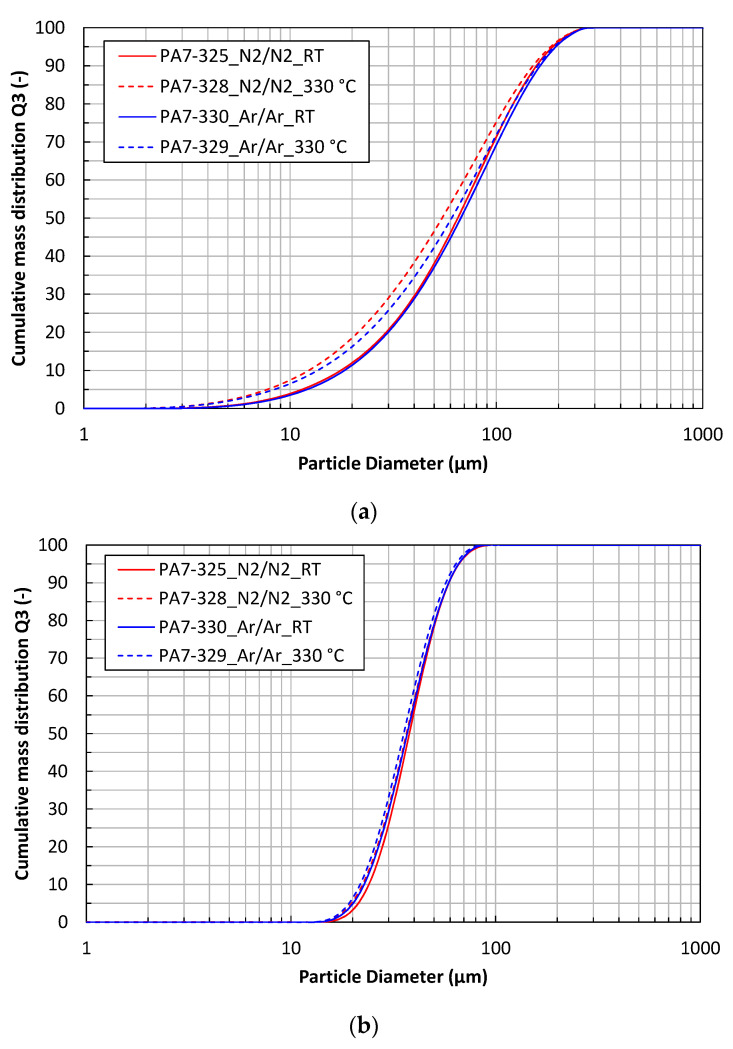
Particle size distributions of the X2CrNiMoCuWN25-7-4 powder atomized with different process gases and gas temperatures. (**a**) Powder fraction < 200 µm; (**b**) powder fraction 20–63 µm.

The cumulative mass distributions (Q3) of the powder particles are shown in Figure 2. The particle size distributions of the steel powders (<200 µm) are significantly affected by the process gas temperature (Figure 2a). Hot gas atomization shifts the particle size distribution to smaller sizes because the gas velocity is considerably increased when it is heated to a high temperature. This results in more efficient fragmentation of the melt and secondary breakup of the ligament and the droplets.

The characteristic particle size parameters d_10_, d_50_ and d_90_ of the powder fractions < 200 µm are given in Table 5. The mass median diameter d_50_ is 65–67 µm for the cold gas atomized powders, while it is 55–61 µm for the hot gas atomized powders. The characteristic particle size parameters of the powder fractions 20–63 µm are given in Table 6. These powders do not show significant differences in their particle size distributions.

The particle shape factors *Circularity* and *Aspect Ratio* were determined based on static image analysis using Morphologi G3 (Malvern Panalytical) [29]. The shape factor *Circularity* is defined as the ratio of the circumference of a circle equal to the particle’s projected *area* to the *perimeter* of the particle:

(6) $$Circularity=\frac{2\sqrt{\pi \cdot Area}}{Perimeter}$$

The shape factor *aspect ratio* represents the elongation of the particle and is defined as the *width*-to-*length* ratio:

(7) $$Aspect\;Ratio=\frac{Width}{Length}$$

Both shape factors are dimensionless and reach a value of 1 for ideal spherical particles.

**Figure 3:**
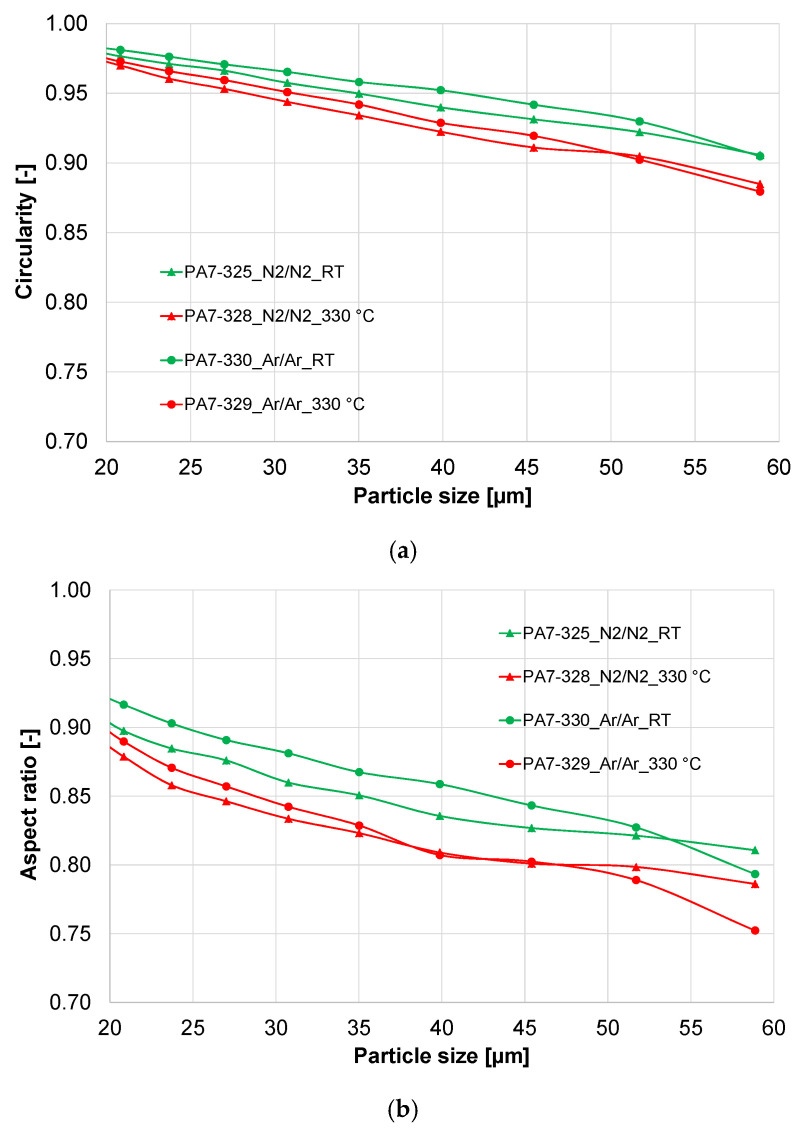
Shape factors of the X2CrNiMoCuWN25-7-4 powder atomized with different process gases (powder fraction 20–63 µm). (**a**) Circularity; (**b**) aspect ratio.

The shape factors of the four powders are presented in Figure 3. The circularity of the powder particles (size 20–63 µm) is in a range of 0.88 to 0.98, with a higher circularity for the smaller particles. The hot gas atomized powders show lower circularity than the cold gas atomized powders. The argon-atomized powders seem to be more spherical than the nitrogen-atomized powders. The aspect ratio of the powders shows the same trend.

The deformation of droplets is governed by the Weber number $We=\frac{d\cdot u_{rel}^{2}\cdot \rho }{\sigma }$, which contains the droplet diameter *d* and the relative velocity *u*_rel_ between gas and droplet. Since the relaxation time of small droplets is much shorter, their relative velocity will be lower during their liquid phase. As a result, small droplets will have a lower Reynolds number during solidification after they have reached their final diameter after several breakups. Accordingly, smaller droplets will usually have a more spherical shape than larger droplets.

Particle collisions may lead to further particle deformation. This is more likely to happen for larger droplets since their solidification duration is longer, and the collision probability increases with droplet size. Since there is a higher fraction of fine particles in the hot gas atomized powders, the collision probability increases resulting in more satellite particles and, therefore, lower circularity and aspect ratio. These aforementioned results are consistent with the reports in the literature [16,20,22].

### 3.3. Particle Morphology

**Figure 4:**
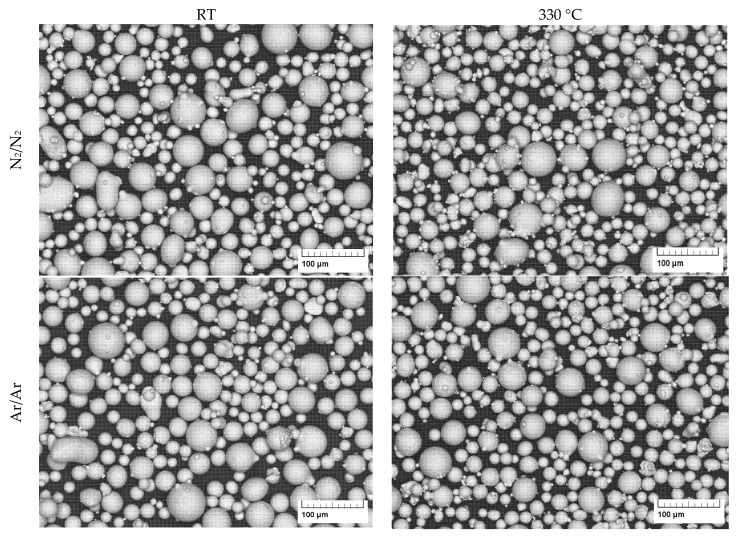
SEM images of gas atomized X2CrNiMoCuWN25-7-4 powders (particle size 20–63 µm, at low magnification).

**Figure 5:**
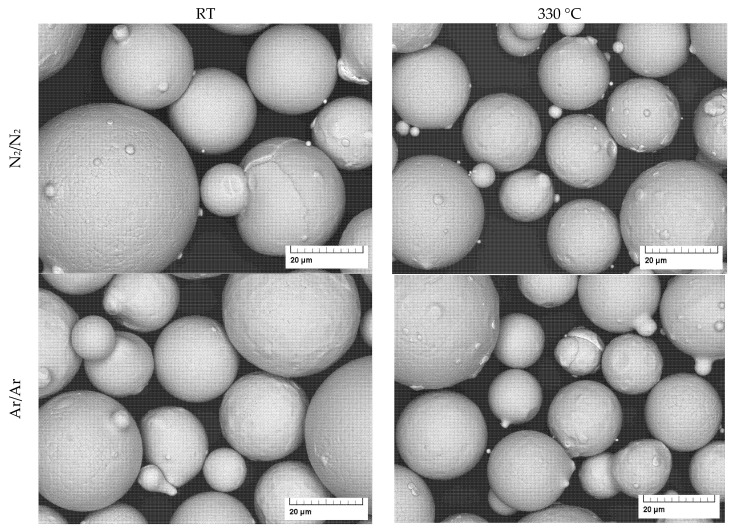
SEM images of gas atomized X2CrNiMoCuWN25-7-4 powders (particle size 20–63 µm, at high magnification).

The micrographs of the gas-atomized powders are presented in Figure 4 and Figure 5. They show a spherical shape with a few satellite particles and typical dendritic structures on the particle surface.

### 3.4. Powder Properties

In general, a high circularity (as well as aspect ratio) results in a good flowability of the powder since the friction and interlocking between the spherical particles are lower than that of the particles in irregular shape [14,15]. However, all the powders in the present study show poor flowability. The avalanche angles of the powders are higher than 50° (see Table 7); meanwhile, their Hausner ratios are in the range of 1.19–1.25. These results also indicate poor/limited flowability of the powders. 

The poor flowability of the powders in the present study could be attributed to the fine particle fraction in the powders. There are about 5% particles smaller than 20 µm in the powders. The fine particles, in combination with their smooth surface, lead to cohesive powders because the van der Waals force between the particles is significantly increased. Although the powders showed poor flowability, they were successfully processed by means of laser powder bed fusion, which will be described in the following section.

### 3.5. Porosity in Gas-Atomized Powder

**Figure 6:**
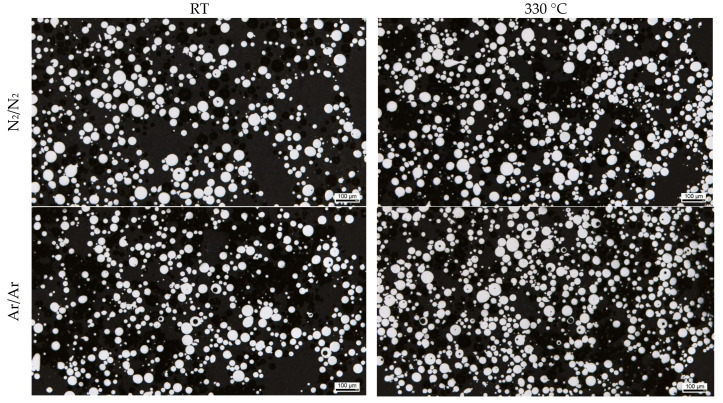
Light micrograph of gas-atomized X2CrNiMoCuWN25-7-4 powders (particle size 20–63 µm, polished sections).

**Figure 7:**
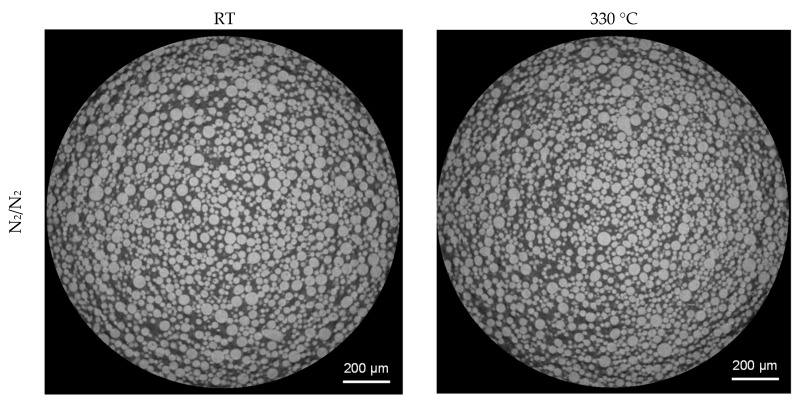

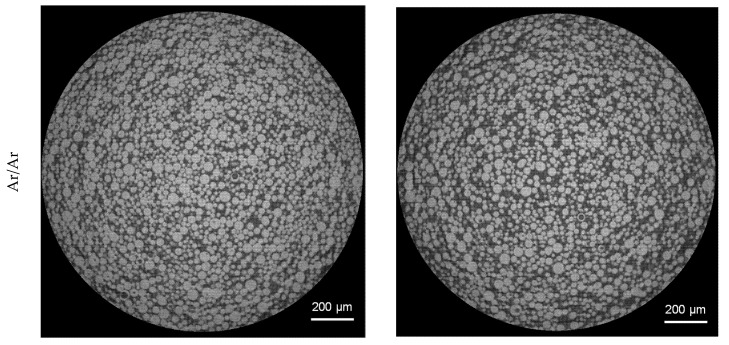
XRM images of gas-atomized X2CrNiMoCuWN25-7-4 powders (particle size 20–63 µm, voxel size 1.56 µm).

The porosity in the powder particles is revealed in the optical micrographs (Figure 6) and in exemplary slices of the XRM scans (Figure 7). It is clearly visible that the powders processed under an argon atmosphere show more hollow particles. The influence of atomization gas temperature on the gas entrapment is not significant according to these results.

**Figure 8:**
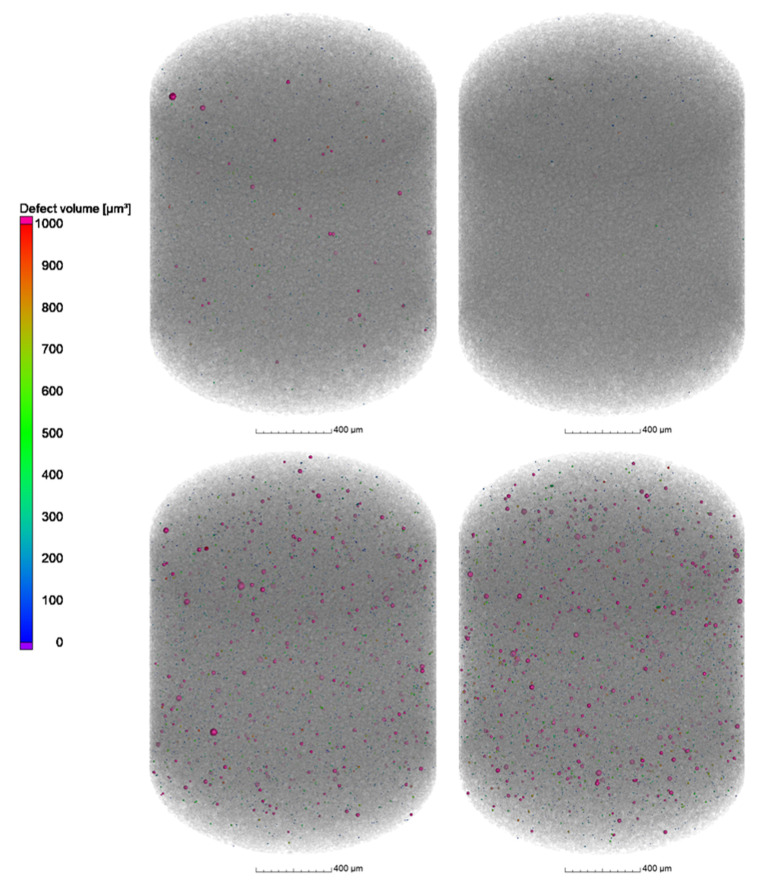
3D-View of porosity of gas-atomized X2CrNiMoCuWN25-7-4 powders (particle size 20–63 µm, voxel size 1.56 µm). Top left: N_2_/N_2_-RT; Top right: N_2_/N_2_-330 °C; Bottom left: Ar/Ar-RT; Bottom right: Ar/Ar-330 °C.

**Figure 9:**
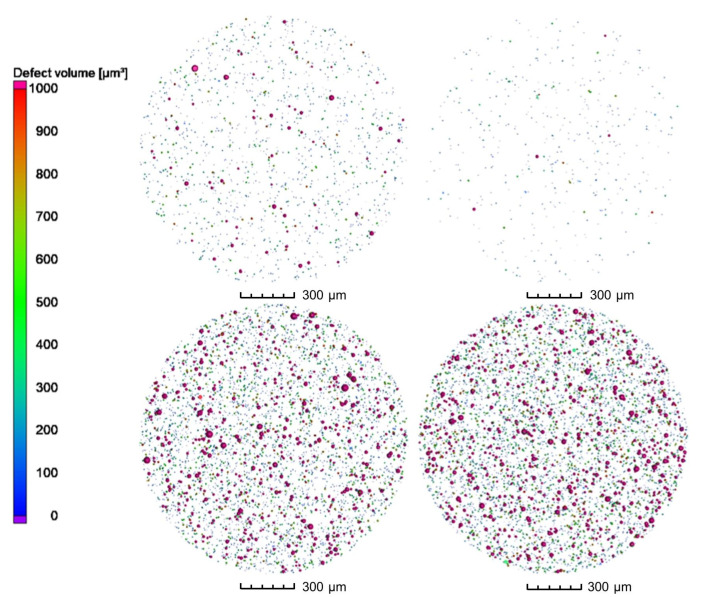
Top view of porosity of gas-atomized X2CrNiMoCuWN25-7-4 powders (particle size 20–63 µm, voxel size 1.56 µm). Top left: N_2_/N_2_-RT; Top right: N_2_/N_2_-330 °C; Bottom left: Ar/Ar-RT; Bottom right: Ar/Ar-330 °C.

3D analysis of the XRM volumes leads to Figure 8, revealing gas pores inside the powder particles. When comparing the powder processed under nitrogen and under argon, it can be clearly seen that a much higher number of pores are present in the particles processed under argon. Both batches processed with nitrogen show a significantly lower number of pores. This is further confirmed by the top view images of the pores in Figure 9. The top view corresponds to a superimposed image of the whole scanned volume, which has approx. 1560 µm in height. By that, especially the reduction of pores can be comprehended easily. Compared to the already small number of pores by processing under nitrogen at RT, it is further decreased when the melt was atomized by hot gas. This effect is not clearly discernable for the pores created in the powder processed under argon. 

**Figure 10:**
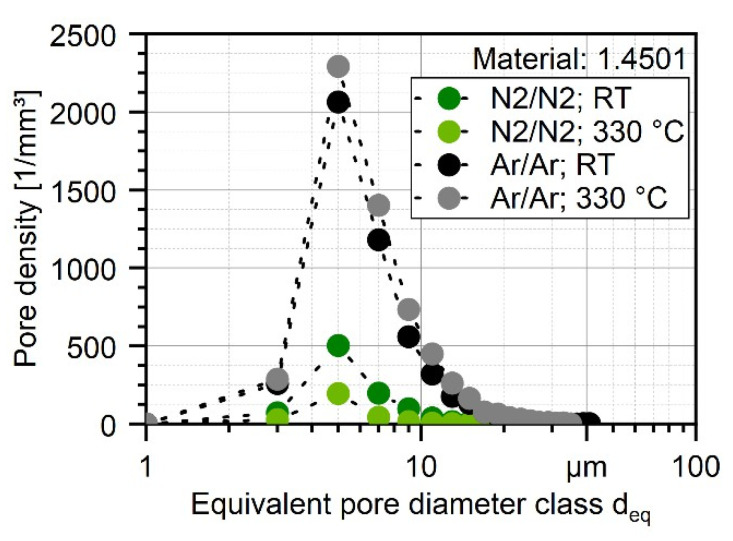
Pore density distribution of nitrogen and argon gas-atomized X2CrNiMoCuWN25-7-4 powders (particle size 20–63 µm, voxel size 1.56 µm) based on XRM measurements.

**Figure 11:**
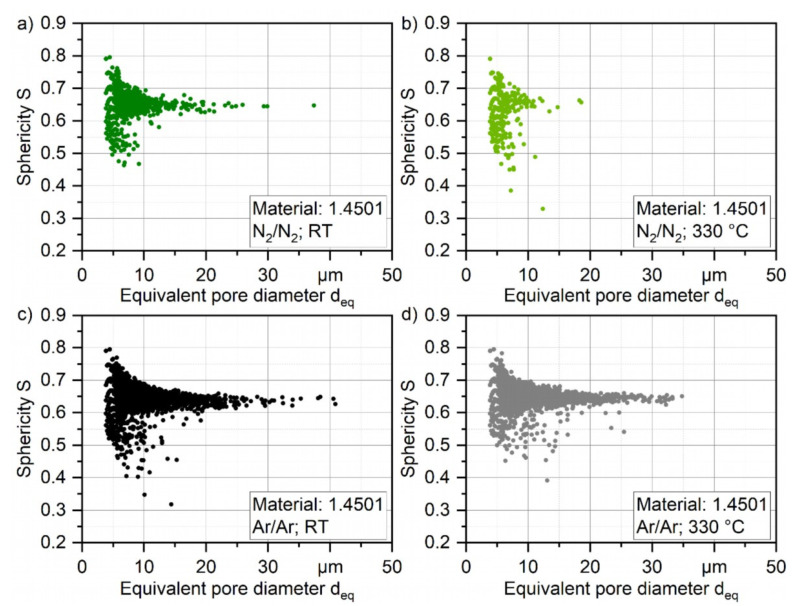
Shape of porosity of gas-atomized X2CrNiMoCuWN25-7-4 powders (particle size 20–63 µm, voxel size 1.56 µm) (**a**) under N_2_/N_2_ at RT; (**b**) under N_2_/N_2_ at 330 °C; (**c**) Ar/Ar at RT; and (**d**) under Ar/Ar at 330 °C.

These results are furthermore supported when plotting the pore density based on the number of pores of each class of *d*_eq_ with a class size of [*d*_eq_ − 1; *d*_eq_ + 1]. The number of pores normalized by the volume of the total scanned material was in the range of 1.4–1.6 mm^3^, as shown in Figure 10. By that, not only the general differences in the number of pores can be quantified, but also the difference in powder particle porosity based on the process parameters can be evaluated. The highest pore density for every processed powder can be found with a size of 4–6 µm for every investigated powder, which is the highest for atomizing with argon at 330 °C (2292 pores per mm^3^) and the lowest with nitrogen at 330 °C (196 pores per mm^3^) which is 88% less. The general difference in porosity is not high and can be found in Table 8, and the porosity is even lower than 0.01% for the N_2_/N_2_ at 330 °C. As shown in Figure 11, the shape of the pores in the powder particles is for all four batches mostly described by sphericity of S = 0.6–0.7. This indicates that the shape is more spherical. In combination with the XRM images in Figure 9, it is safe to conclude that during the atomization of the powder, the process gas is entrapped in the powder leading to the visible and measurable spherical pores inside the particles. Furthermore, atomization in argon gas leads to significantly bigger pores up to a size of *d*_eq_ = 41 µm (Ar/Ar-RT) and 35 µm (Ar/Ar-300 °C). Although for the powder which was atomized under N_2_/N_2_-RT a maximum pore size of *d*_eq_ = 37 µm was found, there were much less pores detected in the range of 20–30 µm as compared for both Ar/Ar batches. The maximum pore size of N_2_/N_2_-330 °C was significantly lower with *d*_eq_ = 18.5 µm.

### 3.6. Porosity in As-Built Cuboids

**Figure 12:**
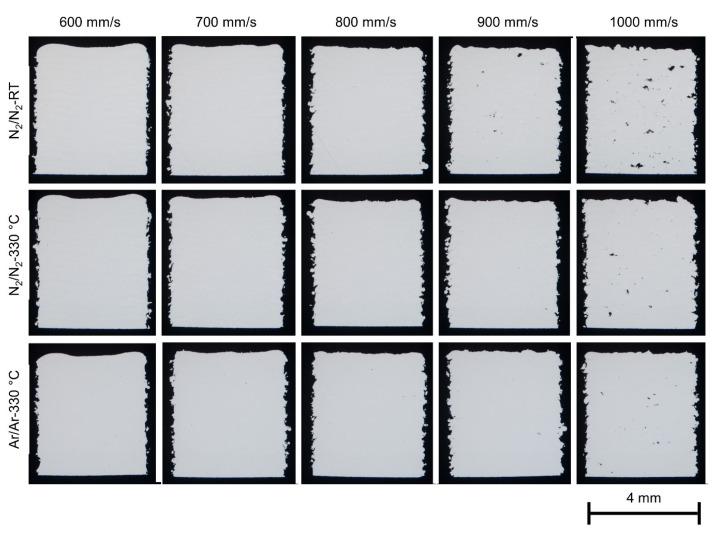
Stereo microscopy of the cubic samples (4 × 4 × 5 mm^3^) built with the various atomized X2CrNiMoCuWN25-7-4 powders (particle size 20–63 µm). The laser power was 250 W for all the samples, and the laser scan speed was varied from 600 mm/s to 1000 mm/s. The building direction is upward. The vertical sections of the samples were ground and polished for optical metallography.

Figure 12 shows the polished sections of the cubic samples built with the various atomized X2CrNiMoCuWN25-7-4 powders. The laser power was set at 250 W for all the samples, and the laser scan speed was varied between 600 mm/s to 1000 mm/s. The PBF-LB/M experiment with the powder PA7-330 (Ar/Ar-RT) was not successful, and the results are not presented in this paper. The duplex stainless steel powders showed good processability during PBF-LB/M in terms of powder spreading (uniform powder layer), remelting and fusion (laser absorption, welding ability). The process window for dense parts is broad for all the powders. Lack-of-fusion was the dominant defect mechanism when the laser energy input was low (at high laser scan speed, for example, 1000 mm/s). 

As aforementioned, the powders show very similar particle size and shape, flowability and powder density. The main differences between the powders are the nitrogen content and the porosity inside the powder particles. The gas entrapment in the powder particles, regardless of the gas chemistry and the gas content, appears to have a negligible effect on the porosity of the as-built parts. The laser parameters (laser energy input) showed a more dominant impact on the porosity of the as-built parts than the powder properties. 

The argon gas entrapped in the powder particles did not lead to increased porosity in the as-built parts. On the contrary, argon gas might be released during PBF-LB/M since the process was carried out under a nitrogen atmosphere. As seen in Table 9, the nitrogen content of the as-built parts printed with the nitrogen atomized powders and the argon atomized powders is 0.39 mass % and 0.13 mass %, respectively. They are slightly higher than that of the gas-atomized powders (0.38 mass % and 0.11–0.12 mass %, respectively). It can be assumed that the outgassing of nitrogen did not occur during the PBF-LB/M, and nitrogen pickup might take place under a nitrogen atmosphere. 

**Figure 13:**
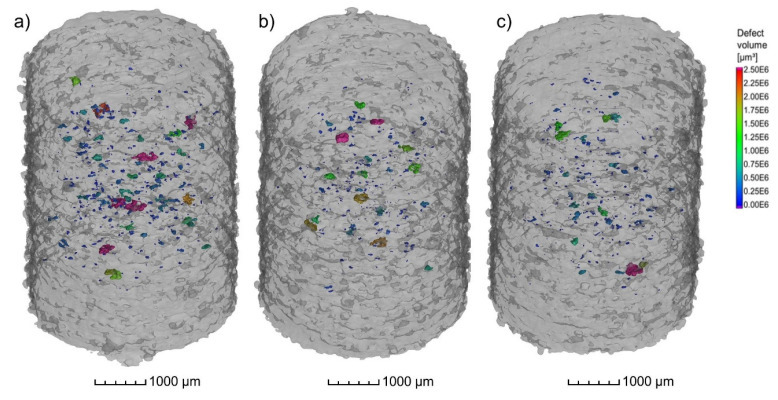
µCT Images of the cylindrical samples (Ø4 × 6 mm^3^) built with the different X2CrNiMoCuWN25-7-4 powders (particle size 20–63 µm, voxel size 7.99 µm) atomized with (**a**) N_2_/N_2_ at RT; (**b**) N_2_/N_2_ at 330 °C; and (**c**) Ar/Ar at 330 °C. The laser power was 250 W, and the laser scan speed was 800 mm/s. The building direction is upward.

The results from µCT investigations on the as-built parts reveal that different powders processed with identical parameters (laser power 250 W and laser scan speed 800 mm/s) lead to only slight differences in porosity and pore distribution. As shown in Figure 13a–c, the highest amount of pores, in combination with the biggest defects, was found in the cylinder manufactured with the powder N_2_/N_2_ at RT (Figure 13a). The porosity in the specimen reached 0.16% whilst the porosity in the specimen manufactured with the N_2_/N_2_ at 330 °C powder (Figure 13b) was about 0.08%. Additionally, the specimen manufactured with the argon-atomized powder (Ar/Ar at 330 °C, Figure 13c) showed an identical porosity of 0.08% with a similar size and position of the pores compared to N_2_/N_2_ at 330 °C. The shape of the pores based on the µCT results indicates that mostly lack-of-fusion defects are present in the specimens. This is concluded based on the sphericity *S* of the defects, which is mostly in the range of 0.4–0.6. As described by Snell et al. [30], these defects can be classified as mostly lack-of-fusion defects. The results also confirm that the porosity in the as-built parts is not directly correlated with the gas entrapment in the powder particles. 

## 4. Conclusions

Compared to argon, the process gas N_2_ in the melting chamber of the atomization plant leads to a higher nitrogen dissolution in the melt of the duplex stainless steel X2CrNiMoCuWN25-7-4 and, therefore, a higher nitrogen content of the atomized powders. 

The duplex stainless steel powder particles typically show a spherical shape and smooth surface, with a few satellite particles. The reduced amount of satellite particles can be traced back to the application of an anti-satellite system within the atomizing chamber. The particle size of the duplex stainless steel powder decreases significantly with increasing the atomization gas temperature. The hot gas atomized particles show lower circularity than the cold gas atomized particles. The argon-atomized particles appear to be more spherical than the nitrogen-atomized particles. The argon-atomized powders have much more gas porosity inside the particles than the nitrogen-atomized powder. 

The duplex stainless steel powders showed good processability during laser powder bed fusion. The process window for dense parts is broad for all the powders. The gas entrapment in the powder particles, regardless of the gas chemistry and the gas content, appears to have a negligible effect on the porosity of the as-built parts. The laser parameters (laser energy input) showed a more dominant impact on the porosity of the as-built parts than the powder properties. Using a nitrogen gas atmosphere could maintain the equilibrium nitrogen content of the powder in the as-built parts.

## Figures and Tables

**Table 1:** Chemical composition of the feedstock determined using S-OES (in mass %).

Alloy	C	Cr	Ni	Mo	Mn	Si	Cu	W	P	S	N	Fe
X2CrNiMoCuWN25-7-4	0.019	25.81	7.73	3.51	0.60	0.22	0.58	0.50	0.022	0.001	0.29	bal.

**Table 2:** Gas atomization parameters of the super duplex stainless steel X2CrNiMoCuWN25-7-4.

Run Number (Exp. ID-Number)	Unit	Run 1 (PA7-325)	Run 2 (PA7-328)	Run 3 (PA7-330)	Run 4 (PA7-329)
Melting atmosphere	-	N_2_	N_2_	Ar	Ar
Atomization gas	-	N_2_	N_2_	Ar	Ar
Atomization gas temperature	°C	RT	330	RT	330
Delivery tube diameter	mm	3	3	3	3
Pouring temperature	°C	1700	1700	1700	1700
Atomization gas pressure	MPa	1.6	1.6	1.6	1.6
Atomization gas flow rate	kg/h	590	393	780	513
Melt flow rate	kg/h	430	404	394	333
GMR (Gas to Melt Ratio)	-	1.37	0.97	1.98	1.54

**Table 3:** Parameters for XRM of the powder particles and µCT scans of the PBF-LB specimens.

Parameters	Unit	XRM	µCT
Acceleration voltage	kV	140	160
Beam current	µA	72	110
Power	W	10.8	17.6
Effective pixel size	µm	1.56	7.99
Exposure rate	ms/fps	354/2.82	250/4.00

**Table 4:** PBF-LB process parameters.

Laser Power (W)	Scan Speed (mm/s)	Laser Spot Size (µm)	Hatch Distance (µm)	Layer Thickness (µm)	Scan Strategy	Rotation between Layers (°)	Process Gas
250	600–1000	50	100	50	Simple hatching	67	N_2_

**Table 5:** Particle size parameters of the powder fraction < 200 µm.

Run Number (Exp. ID-Number) Process Gas		Run 1 (PA7-325) N_2_/N_2_-RT	Run 2 (PA7-328) N_2_/N_2_-330 °C	Run 3 (PA7-330) Ar/Ar-RT	Run 4 (PA7-329) Ar/Ar-330 °C
d_10_	µm	23.9	22.4	22.7	21.7
d_50_	µm	65.1	54.6	67.3	60.8
d_90_	µm	156.5	151.0	163.7	158.8

**Table 6:** Particle size parameters of the powder fraction 20–63 µm.

Run Number (Exp. ID-Number) Process Gas		Run 1 (PA7-325) N_2_/N_2_-RT	Run 2 (PA7-328) N_2_/N_2_-330 °C	Run 3 (PA7-330) Ar/Ar-RT	Run 4 (PA7-329) Ar/Ar-330 °C
d_10_	µm	24.0	22.4	22.8	21.8
d_50_	µm	37.9	36.7	37.0	35.6
d_90_	µm	59.1	59.0	59.0	57.1

**Table 7:** Powder properties (powder fraction 20–63 µm).

Run Number (Exp. ID-Number) Process Gas		Run 1 (PA7-325) N_2_/N_2_-RT	Run 2 (PA7-328) N_2_/N_2_-330 °C	Run 3 (PA7-330) Ar/Ar-RT	Run 4 (PA7-329) Ar/Ar-330 °C
Avalanche angle	°	57.5	54.9	53.6	53.6
Apparent density	g/cm^3^	3.98	3.82	3.95	3.87
Tap density	g/cm^3^	5.00	5.00	4.76	4.76
Hausner Ratio	-	1.25	1.25	1.19	1.22

**Table 8:** Porosity of the different powder batches based on XRM.

Run Number (Exp. ID-Number)	Process Gas	Porosity (in %)
Run 1 (PA7-325)	N_2_/N_2_-RT	0.03
Run 2 (PA7-328)	N_2_/N_2_-330 °C	<0.01
Run 3 (PA7-330)	Ar/Ar-RT	0.14
Run 4 (PA7-329)	Ar/Ar-330 °C	0.21

**Table 9:** Chemical composition of the as-built parts determined using S-OES (in mass %).

Powder Number (Exp. ID-Number)	C	Cr	Ni	Mo	Mn	Si	Cu	W	P	S	N	Fe
PA7-325 (V417)	0.031	25.22	7.71	3.36	0.53	0.25	0.61	0.54	0.025	<0.001	0.393	bal.
PA7-328 (V418)	0.032	25.26	7.69	3.33	0.53	0.25	0.58	0.54	0.024	<0.001	0.396	bal.
PA7-329 (V419)	0.020	25.33	7.68	3.31	0.53	0.26	0.60	0.54	0.026	<0.001	0.138	bal.

## Data Availability

Data from the study presented can be received from the corresponding author upon request.
